# Symptom reduction in mal de débarquement syndrome with attenuation of the velocity storage contribution in the central vestibular pathways

**DOI:** 10.3389/fresc.2024.1331135

**Published:** 2024-02-29

**Authors:** Jun Maruta, Catherine Cho, Theodore Raphan, Sergei B. Yakushin

**Affiliations:** ^1^ Department of Neurology, Icahn School of Medicine at Mount Sinai, New York, NY, United States; ^2^ Department of Rehabilitation and Human Performance, Icahn School of Medicine at Mount Sinai, New York, NY, United States; ^3^ Department of Neurology, NYU Langone Medical Center, New York, NY, United States; ^4^ Department of Otolaryngology, NYU Langone Medical Center, New York, NY, United States; ^5^ Department of Computer and Information Science, Brooklyn College, Institute for Neural and Intelligent Systems, New York, NY, United States; ^6^ The Graduate School and University Center of the City University of New York, New York, NY, United States

**Keywords:** aging, central vestibular disorder, gravity, orientation vector, rocking, swaying, bobbing, non-spinning vertigo

## Abstract

**Background:**

The velocity storage mechanism of the central vestibular system is closely associated with the vestibulo-ocular reflex (VOR), but also contributes to the sense of orientation in space and the perception of self-motion. We postulate that mal de débarquement syndrome (MdDS) is a consequence of inappropriate sensory adaptation of velocity storage. The premise that a maladapted velocity storage may be corrected by spatial readaptation of the VOR has recently been translated into the development of the first effective treatment for MdDS. However, this treatment's initial impact may be reversed by subsequent re-triggering events. Presently, we hypothesized that MdDS symptoms could alternatively be reduced by attenuating the velocity storage contribution in the central vestibular pathways.

**Methods:**

Forty-three patients with MdDS (aged 47 ± 14 yo; 36 women) were randomly assigned to two treatment groups and followed for 6 months. The horizontal VOR was tested with chair rotation during laboratory visits, and the strength of velocity storage was quantified with model-based parameters—the time constant (Tc) and the gain of coupling from the vestibular primary afferent signals (g_0_). To attenuate velocity storage, Group 1 underwent a progressively intensifying series of low-frequency earth-vertical oscillatory rotation coupled to conflicting visual stimuli. Group 2 underwent an established protocol combining head tilts and visual stimulation, designed to correct maladapted spatial orientation but not change the velocity storage strength. The symptom severity was self-rated on an 11-point scale and reported before and up to 6 months after the treatment.

**Results:**

In Group 1, velocity storage was modified through reduction of g_0_ (*p* < 0.001) but not Tc. The symptom rating was at least halved initially in 43% of Group 1 (*p* = 0.04), the majority of whom retained a similar level of improvement during the 6-month follow-up period. In Group 2, no systematic change was induced in the parameters of velocity storage strength, as expected. The symptom rating was at least halved initially in 80% of Group 2 (*p* < 0.001), but paralleling previous findings, symptoms often returned subsequently.

**Conclusion:**

Attenuation of velocity storage shows promise as a lasting remedy for MdDS that can complement the VOR readaptation approach.

## Introduction

Mal de débarquement syndrome (MdDS) is considered a rare illness but nevertheless counted among common balance disorders (1). MdDS, which is typically triggered by prolonged exposure to passive motion during a voyage on a cruise ship or airplane, is primarily characterized by a continuous perception of oscillatory self-motion such as rocking, swaying, or bobbing, or a sensation of gravitational pull (collectively identified as non-spinning vertigo) and associated sensations of imbalance (2–4). The self-motion symptoms of MdDS are typically accompanied by somatic complaints (e.g., headaches and visually induced dizziness), reduced cognitive function (e.g., decreased attention and short-term memory), and affective problems (e.g., depression and anxiety). These symptoms can be severe enough for some patients to develop suicidal thoughts and often lead to long-term disability.

Although mal de débarquement, i.e., a transient illusion of self-motion following exposure to prolonged passive motion, has been recognized for centuries (5, 6), and its chronic manifestation, MdDS, has attracted increasing interest in the wake of a 1987 publication of a six-patient case series (2), MdDS still has not permeated the awareness of clinicians. MdDS is often misdiagnosed as a mental disorder, vestibular migraine, or peripheral vestibular dysfunction, and patients on average make 19 (but more typically 2–5) visits to healthcare professionals before their MdDS diagnosis (3, 7–9). Given these circumstances, it is presently not possible to determine the actual prevalence of the illness. However, MdDS may represent at least a small percentage of patients seen at large clinical centers specializing in balance and dizziness (10, 11) and reportedly has a strong female predominance of 80%–90% (9, 12, 13).

The number of people seeking treatment is expected to increase because general awareness of the illness is improving—according to our patients' intake forms, most patients self-diagnose for MdDS over the Internet first, and then confirm their diagnosis with specialists. Furthermore, cruises were one of the fastest-growing tourism industries before the COVID-19 pandemic, growing from 17.8 million passengers worldwide in 2009 to 29.7 million in 2019 (14). At the time of this writing, full recovery of the industry was projected in 2023 (15).

However, treatment options for MdDS are limited. In fact, until recently the illness was considered intractable, with a progressively lower likelihood of remission as time passed (3). Conventional vestibular physical therapy is generally ineffective in treating MdDS (13, 16, 17). Benzodiazepines, a class of GABA-A agonists, may provide partial symptom relief for some patients (13, 16, 18), but if effective, the site of its action is not understood, and harmful effects including dependence must be considered (19, 20). Treatment with vestibular migraine medications can improve the quality of life of patients with MdDS, but symptom improvement appears domain-specific, and a greater degree of dose management than typical may be required due to their sensitivity to medications (21, 22). Alternatively, studies have suggested that disrupting the inappropriate entrainment in a neural functional-connectivity network using non-invasive brain stimulation methods during a span of days may reduce symptoms (23–25). However, the long-term outcome of this treatment is unknown.

In contrast to these symptom-focused approaches, the recent discovery that MdDS may involve maladaptation of the velocity storage mechanism of the central vestibular system opened opportunities for positive long-term outcomes by addressing the root cause of the illness (12, 26, 27). Velocity storage is activated by head rotation, large-field visual motion, or proprioceptive cues for continuous rotation, and temporarily holds, or stores, an estimate of head rotational velocity in space (28–32). The velocity storage mechanism is thought to support spatial orientation by acting as a “neural gyroscope” (33–35). Velocity storage is closely associated with the vestibulo-ocular reflex (VOR) as it was first examined as a stored eye movement drive related to head rotation during vestibular and optokinetic nystagmus (29–31, 36), but is also thought to contribute to postural control (37, 38) and the perception of spatial orientation and self-motion (28, 30, 31, 39).

An animal-based study showed that spatial orientation properties of velocity storage could be maladapted by exposure to unnatural motion, as revealed in the consequent abnormal VOR (40). It was thus postulated that dysfunction of velocity storage, particularly in the form of misaligned spatial orientation, could cause primary symptoms and signs of MdDS. Based on this postulate, a treatment protocol was subsequently designed to correct such misalignment by stimulating readaptation of the VOR through exposure to full-field visual motion coupled with head tilts at the frequency of the phantom oscillation (26). Support for the postulate comes from the clear effect subjectively reported by the majority of over 600 patients treated with the VOR readaptation protocol in our laboratory thus far (12, 26, 27). It is unlikely that this effect was due to spontaneous recovery or a placebo response because of the chronicity of MdDS in these patients, who on average, had had the illness for two years before receiving the readaptation treatment and approximately 5% for as long as more than a decade (up to 41 years). The protocol yielded an overall strong positive initial impact even among patients with long durations of the illness even though other treatments had been sought priorly. This method's effectiveness has been independently confirmed by others (41–44). Further support for the velocity storage involvement in MdDS comes from a sham-controlled study, which demonstrated a treatment effect when head tilts were coupled with large-field visual motion as in the original protocol but not with a non-moving but otherwise identical visual pattern (45). A recent sequel animal-based study also supports that the effect of VOR maladaptation can be systematically cumulated or reversed by the choice of the vestibular stimulus (46). Together, VOR readaptation has come to be recognized as the first effective treatment for MdDS (47).

Unfortunately, while overall significant improvement in MdDS outcomes has been attained with VOR readaptation, about 25% of patients have been found not to benefit from this method, and re-exposure to prolonged passive motion or provocative visual stimuli can reverse the initial benefit after a successful treatment (12, 26). For these patients, an alternative approach is needed, particularly in delivering a countermeasure against provocative motion and visual stimuli. Velocity storage provides a critical control point for this purpose as well given its key role in visual-vestibular integration (29–31).

**Figure 1:**
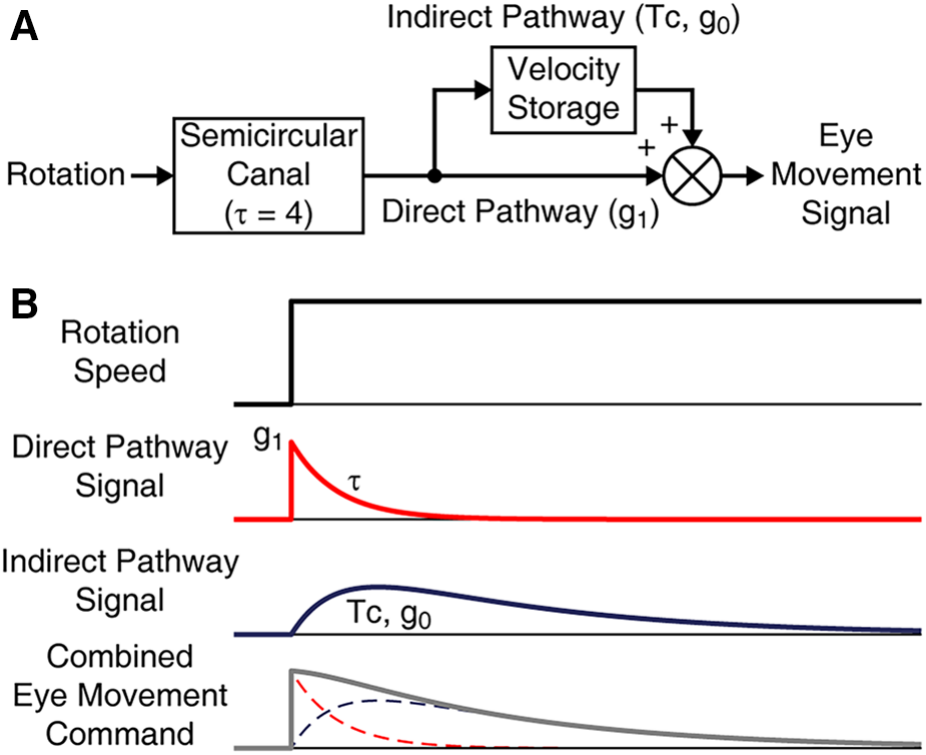
Model-based characterization of the VOR in darkness. (**A**) The VOR ideated as a combined output of direct and indirect pathways. The peripheral response to rotation has a time constant (*τ*) of 4 s. The direct pathway is in addition associated with a gain parameter g_1_. The indirect pathway is associated with Tc and g_0_. (**B**) Temporal response profiles of the model elements in reaction to a rotational velocity step stimulus.

Velocity storage is most conventionally characterized with its activation during the VOR in darkness. In particular, the VOR slow phase velocity profile can be modeled as the sum of the outputs of the velocity storage and non-velocity storage pathways, in which the latter directly reflects the well-characterized peripheral vestibular activity (Figure 1A) (30, 48). In response to a velocity step rotation about a spatial vertical axis, the peripheral activity suddenly rises and then decays exponentially, the time constant for which is relatively invariant across individuals and estimated to be ≈4 s (Figure 1B, Direct Pathway Signal) (48, 49). The gain of the direct pathway (g_1_) corresponds to the gain of the initial rise in the slow phase velocity of the VOR nystagmus. The velocity storage component can then be profiled in terms of its rate of charge/discharge, i.e., time constant (Tc), and the strength of connection with the direct pathway, i.e., gain of coupling (g_0_), estimated from the model-based fit of the VOR slow phase velocity profile (Figure 1B, Indirect Pathway Signal). The present study does not include a characterization of the three-dimensional behavior of velocity storage. Although a three-dimensional articulation of VOR parameters expanded with cross-axis coupling terms has been formulated to express the spatial orientation properties of velocity storage (34, 50, 51), pertinent parameter estimation demands three-dimensional eye movement recording with appropriate test paradigms. This limitation has made it difficult to directly demonstrate the effect of the VOR readaptation treatment in terms of a change in the spatial orientation properties of velocity storage.

Age is among the various factors for inter- as well as intra-individual variations in velocity storage characteristics—the Tc reportedly is short in infants, increases through young adulthood, and then gradually decreases with aging (52, 53). Velocity storage can be modified in an individual—repeated vestibular stimulation can shorten the duration of the VOR with a diminished contribution of velocity storage in an effect known as habituation (54, 55), interpreted as shortening of the Tc (56–58). Some reports indicate fighter jet pilots, ballet dancers, and figure skaters are habituated to vestibular stimuli, although others question such generalization (59–64). Curiously, while velocity storage, as a center of multimodal sensory integration, may be useful in some contexts (28–32), habituated individuals show no known functional impairment.

Presently, we hypothesized that, if MdDS is caused by malfunctioning velocity storage, attenuating its contribution through reduction of Tc or g_0_ will reduce the symptoms of MdDS. Velocity storage can be safely and greatly attenuated within 4–5 days using a protocol previously developed in our laboratory to reduce susceptibility to motion sickness (58). The new approach would be complementary to VOR readaptation, the latter of which aims to correct the spatial orientation properties of velocity storage rather than to change Tc or g_0_. Moreover, since both animal- and human-based research suggests long-term retention of velocity storage attenuation (55, 57, 58, 65), we further hypothesized that this new utility would yield robust long-term outcomes. Thus, in this exploratory study, we set out to contrast the effects of the velocity storage attenuation and VOR readaptation regimens in the treatment of patients with MdDS and to elucidate how these approaches might be able to complement each other.

## Methods

### Patient selection

The study protocol was reviewed and approved by the Institutional Review Board of Icahn School of Medicine at Mount Sinai. Patient volunteers with MdDS were recruited through various sources of referral and announcements posted on the Internet, including ClinicalTrials.gov (NCT04213079). Applicants seeking treatment were screened with an intake form, and each candidate's diagnosis of MdDS with an associable motion trigger was confirmed by a board-certified physician through a telephone interview when necessary. The accuracy of the paperwork was then verified again. The eligibility criteria were similar to those in our previous studies (12, 27, 38): (1) presentation of continuous oscillatory vertigo and/or gravitational pulling, which had persisted for at least 3 weeks; (2) symptom onset within 48 h after exposure to prolonged passive motion; (3) improvement in symptoms when in a moving vehicle (e.g., a car) and a return of symptoms with the stop of the vehicle (4); (4) No history of head or neck trauma, Lyme disease, serious peripheral vestibular disease, or other major neurological disorders; (5) normal nystagmography reports; and (6) 18–78 years of age. Many had completed neurologic and otologic workups, including magnetic resonance imaging, that were unremarkable. Applicants were informed that, if selected, they would be randomly assigned to one of the two treatments. As an incentive to remain in the study, applicants were also informed that after completing a six-month post-treatment follow-up, they would have an opportunity to return to the laboratory and receive the same or alternative treatment for free of charge if the symptoms persisted or returned. Enrolled subjects were asked to stay in the New York Metropolitan area during the treatment period to minimize the risk of exposure to passive motion that might confound the effect of the treatment. None of the subjects were taking a benzodiazepine medication regularly during the study participation.

### Self-reported MdDS symptom severity

The manifestation of MdDS is often only subjective, and thus, the severity of the illness cannot be judged with physical signs. As with our previous studies (12, 27), the overall severity of MdDS-related symptoms, including not only the sensation of self-motion but also somatic, cognitive, and affective problems, was subjectively reported on a single 11-point scale of 0–10, where the score 0 indicated no symptoms and 10 the most difficult of combined symptoms that the patient subject could imagine. This self-rating was used to document the presence or absence of symptoms and changes in subjective perception of their overall severity, and to assess treatment effects for a particular subject rather than to compare symptom severity between individuals. Subjects were asked to report their symptom severity before and immediately after the treatment, as well as at 2-week, and 1-, 3-, and 6-month follow-ups. This measure was the primary outcome examining the treatment regimens' efficacy for symptom reduction.

### Nystagmography

Subjects were tested for their VOR while seated in a rotational chair in a closed cylindrical chamber that had an inner radius of 90 cm (Neurokinetics, Pittsburg, PA). Eye movement was recorded with videooculography at a rate of 60 frames per second (Model RK-416, ISCAN, Woburn, MA) or 240 frames per second (FN-VN-02-240B, FNND LLC, Elmwood Park, NJ) and calibrated by having the subject look at a laser-projected red dot on the wall of the darkened chamber. The VOR of one subject from Group 2 could not be tested due to claustrophobia. Eye movement of another subject from Group 2 could not be recorded due to equipment malfunction. The eye movement of an additional subject from each group could be recorded successfully only on the first day, similarly due to equipment malfunction.

To screen for a possible cerebellar abnormality, the ability to suppress the VOR with a visual cue was tested on the first day of the subject's laboratory visit. This test was conducted with sinusoidal side-to-side rotation about a spatial vertical axis at 0.1 Hz with a peak speed of 60°/s, first in complete darkness and then with a laser-projected red dot that moved with the chair, i.e., stationary relative to the subject in motion. To characterize and contrast the vestibular physiological responses to the treatment regimens, the VOR was tested with a velocity-step rotation about a vertical axis on each day of the laboratory visit before the day's treatment regimen. The VOR was characterized with daily pre-treatment assessments because expression of velocity storage depends on the levels of fatigue and alertness of the subject (66–69). The test was conducted by accelerating the chair in darkness from 0 to 60°/s within 200 ms, holding the velocity until the induced per-rotatory nystagmus dissipated, and decelerating the chair to stop within 200 ms. After the post-rotatory nystagmus dissipated, the direction of the rotation was reversed.

**Figure 2:**
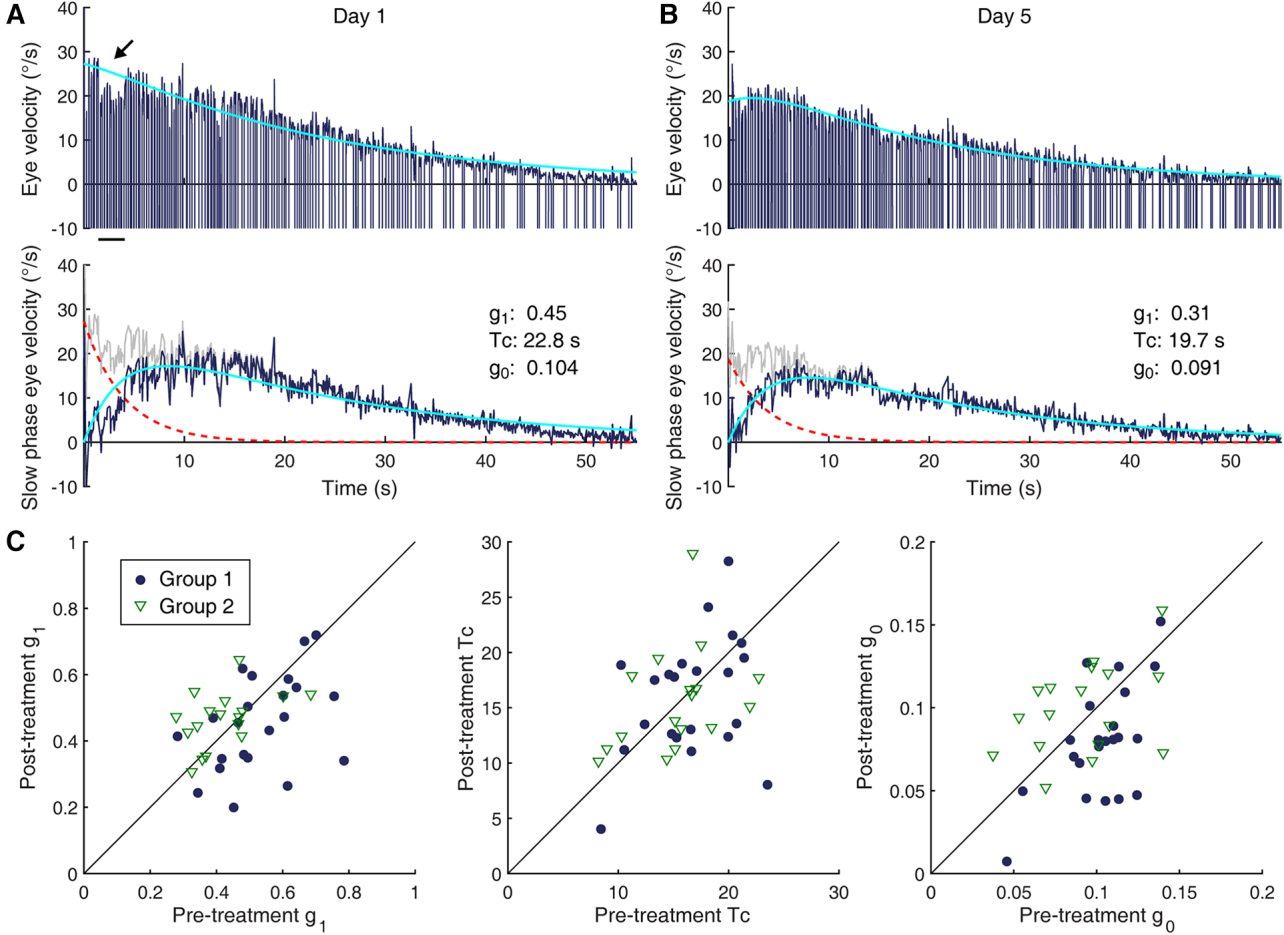
Changes in the VOR. (**A,B**) Characterization of VOR responses. Shown are per-rotatory eye velocity responses to a leftward 60°/s rotation by a 58-year old woman (Subject 16, Group 1) on Day 1 (**A**) and Day 5 (**B**) Top row: eye velocity profile of the nystagmus (dark blue) and a double exponential model-based fit of the slow phase modulation (cyan). Fast phase velocity profiles are truncated at −10°/s. The dip in the eye velocity profile indicated by the arrow corresponds to low-amplitude, high-frequency oscillations (shimmering nystagmus), not reflective of an actual reduction of the slow phase eye velocity. Second row: decomposition of the model elements—slow phase modulation profile (gray), direct pathway contribution (dashed red trace), velocity storage contribution (dark blue), and model fit (cyan). (**C**) Pre- and post-treatment measures of VOR parameters in individual subjects (Group 1: filled circles; Group 2: open triangles). (Left) g_1_. (Center) Tc. (Right) g_0_. The diagonal lines indicate lines of equality.

Data were processed using a software program developed in our laboratory (70). Saccades in eye velocity traces were identified using an order-statistic filter (71), followed by visual inspection and manual correction, and replaced with straight lines connecting the remaining segments. For the VOR suppression test, the horizontal slow phase velocity profiles were fit with sine functions to assess the percentage of response reduction. Visual suppression of the VOR by more than 85% was considered normal. For the velocity-step test, the horizontal slow phase velocity profile was fit with a double exponential curve, for which the first exponent was constrained with the initial peak velocity and a time constant of 4 s representing the semicircular canal response with g_1_ denoted as the direct pathway gain, and g_0_ and Tc were derived as the second component representing the velocity storage response (Figures 1, 2A,B) (48). The values of g_1_, g_0_ and Tc computed from two per-rotatory and two post-rotatory responses from right- and leftward rotations were then averaged to reduce statistical noise due to random performance variability. Linear regression was used to determine the trend of each VOR parameter's changes over days of treatment within individuals. The ordinate intercept and the trendline value corresponding to the last day of treatment were considered to represent the pre- and post-treatment values, respectively. For the two subjects whose eye movement were recorded only on the first day of their laboratory visits, only the pre-treatment values represented by these data were considered.

A large data set of Tc and g_0_ was available through previous clinical and research testing for velocity storage characteristics conducted in our laboratory. We reviewed 6,065 de-identified records made between 1993 and 2019. Data were selected as non-MdDS with normal vestibular function if they were part of a study with normal subjects, or taken from patients with a complaint of dizziness due to quick changes in body orientation such as standing after a long period of sitting or lying down (orthostatic intolerance) or from patients who came to the laboratory for testing after being treated for benign paroxysmal positional vertigo. We identified 911 such records (571 women; 340 men, age range: 13–95 years old). We also identified similar previous records of 28 patients diagnosed with MdDS (25 women, 3 men), not overlapping with the current cohort. Age, sex, Tc, and g_0_ data were extracted from these records for analysis. The data from these historical cohorts together with those from the current MdDS cohort were used to examine potential abnormalities in Tc or g_0_ in patients with MdDS.

### Assessment of vestibular imbalance and posture

The internal sensation of motion or imbalance in MdDS is often not manifested as a physical sign (12). However, the Fukuda stepping test (72) and static posturography were routinely conducted to supplement the subjects’ verbal description of their sensations. The results were also used to guide the stimulus parameters for the VOR readapation treatment (Group 2) as described below. The subject performed the Fukuda stepping test on the first day of the laboratory visit before the treatment. Posturography was conducted each day and to supplement verbal feedback. Postural data were recorded using a Wii board (Nintendo Co. Ltd. Kyoto, Japan), whose output was sampled at 10 Hz and cubic-spline upsampled to 1,000 Hz (12). Posture was assessed with the feet ≈30 cm apart with the eyes open and closed, and the feet together with the eyes closed. To register the direction and frequency of the subjective sensation of self-motion, subjects were often asked to move their bodies while standing on the Wii board in a manner that exaggerated what they felt. The dominant frequencies of the postural instability in the sagittal and coronal planes were determined from the power spectra of the recorded center of pressure (73).

### Group 1—treatment with velocity storage attenuation

To attenuate velocity storage, a conflict was induced between two velocity storage-mediated responses, namely optokinetic nystagmus (OKN) and the VOR during sinusoidal side-to-side rotation about a spatial vertical axis at low frequency. Following the previously described protocol that induced vestibular habituation with shortened Tc, 0.017 Hz (i.e., 60 s period) was used (58). The VOR of normal individuals at this frequency on average reportedly has a phase advance of approximately 30° and a gain of 0.68 relative to the ideal compensatory response (58). In contrast, the slow phase eye velocity of the OKN at such a low frequency has no phase advancement relative to the optokinetic stimulus (OKS) (74). A full-field horizontal OKS was generated by projecting vertical stripes against the wall of the cylindrical enclosure from a projector rotating about a vertical axis directly above the subject's chair. The width of the stripes was 8 cm for the projected light and 11 cm for the interposed shadows, respectively corresponding to 5° and 7° in visual angle. To simplify the protocol, a VOR gain of 0.68 and a phase advance of 30° relative to the ideal were assumed across all subjects, and the OKS was set 180° out of phase with the expected VOR to have OKN counteract it (58).

Since the conflict stimulus was expected to be overwhelming to subjects at high speeds, they were first trained with a peak rotation speed of 5°/s, which was gradually increased over days up to 50°/s. This speed was higher than the 20°/s benchmark used in the treatment of patients with high susceptibility to motion sickness (58). When the protocol was previously clinically applied to MdDS, patients began to show signs of symptom improvement when the peak rotation speed reached 30–40°/s; therefore, 40°/s was considered a benchmark of treatment completion in the present application, although two subjects were unable to tolerate 40°/s by the last day of the treatment. Each training session was targeted to last for 20 min, with a 10-min break provided between sessions. Two to three sessions were administered each day for a total duration of ≈300 min, typically completed in five days. To stay alert, subjects were encouraged to listen to an audio program of their choice during the session. However, a full-field OKS is so powerful that one would not need to be attentive to the moving stripes to experience vection from the visual motion. The brightness of the OKS projector was adjustable. Based on our previous experience, a brightness of 2 lux was deemed tolerable to most patients with MdDS. Thus, the projector brightness was initially set to 2 lux. One subject could not tolerate the initial setting, and the training was resumed with a peak rotation speed of 2°/s, brightness of 1 lux, and duration of 5 min. However, this subject was able to complete the treatment protocol by Day 5 with a 50°/s peak rotation speed, brightness of 2 lux, and duration of 20 min. For another subject who reported no discomfort and had no history of migraine or motion sickness, the brightness was increased to 3 lux from Day 3. In case of nausea or other elevated signs of motion sickness, the treatment was discontinued until the next day.

### Group 2—treatment with VOR readaptation

To induce a change in the spatial orientation properties of velocity storage, a full-field, unidirectional horizontal OKS was generated in the cylindrical enclosure and combined with a head maneuver (12, 26). This stimulus was not presumed to change g_1_, Tc, or g_0_. The combination of the OKS and the head maneuver was customized for each subject based on the phantom motion sensations experienced by the subject. The stimulus was further customized to minimize side effects from overexposure to OKS, such as head pressure, brain fog, fatigue, and migraine, which were anticipated due to an elevated sensitivity to moving visual stimuli in many patients with MdDS. The initial duration of the treatment session, OKS velocity, and projector brightness were respectively set at a mild level of 1 min, 5°/s, and 2 lux based on our previous study (12). When subjects reported no discomfort with the stimulus while reporting no or negligible improvement in symptoms, the duration of the treatment session was increased up to 10 min, the OKS velocity up to 10°/s, and projector brightness up to 3 lux, as tolerated.

The OKS direction was chosen to oppose the direction indicated by the Fukuda test or that of the sensation of pull or circular body motion (12, 26). In the absence of such indications, the direction was chosen arbitrarily. The head maneuver was orthogonal to the motion sensation or the postural instability of the subject. Thus, when the motion was characterized mainly as rocking back-and-forth, the head was rolled from side to side about the naso-occipital axis. When the motion was characterized mainly as swaying from side to side, the head was pitched forward and backward about the interaural axis. The frequency of head tilts was chosen to approximate the frequency determined from the posturography measures, which typically was expected to be near 0.2 Hz (26, 75). The magnitude of head tilts was initially ≈±20° but was varied from ≈±5° to ≈±30° depending on the subject's response to the treatment. The choice of the OKS direction was tested with a 1-min administration of the stimulus combined with a head maneuver. If symptom improvement was reported, the treatment was continued at half the initial frequency of head motion for 2 min and then at a quarter of the initial frequency for 3 min. A ≈5 min break was given between trials.

After completing the sequence, subjects would often report substantial immediate improvement in their symptoms. In such cases, we asked the subjects to go outside for 10–15 min to expose themselves to the naturally busy visual environment of New York City streets. When symptoms were re-triggered, the treatment sequence was repeated, but otherwise, no further treatment was given that day.

When worsening or no improvement of symptoms was reported with the initial choice of the OKS direction, the direction was reversed. When no improvement was reported for either direction, the treatment duration was increased to 2 min without changing the head maneuver frequency. When still no improvement was reported, the stimulus was intensified by increasing the projector brightness and/or the OKS speed, but without changing the OKS direction. The treatment, with breaks, was continued during the allocated time for the day's visit of 90 min, unless the subject reported discomfort such as head pressure and headache.

When the subject reported improvement of symptoms on the next day, the protocol used on the previous day was repeated. When the subject reported worsening or no changes in symptoms, the opposite OKS direction was applied. Additionally, we found that some subjects responded well only when the head was oscillated at a specific frequency. For these subjects, the total duration of treatment sessions at that frequency was increased.

### Statistical analysis

Group characteristics were compared with a Fisher exact test (sex), a two-sample *t*-test (age and VOR parameters), or a Wilcoxon–Mann–Whitney test (MdDS duration). For each group, within-subject changes associated with the respective treatment intervention in the VOR parameters (g_1_, g_0_, and Tc) were tested with a paired *t*-test. The alpha level was set at 0.05. The effect size of a difference between two means or a deviation of the mean from zero in the VOR parameters was examined with a coefficient d, defined as the mean difference divided by the corrected sample standard deviation.

A trend in a scatter plot of Tc or g_0_ data from the non-MdDS historical cohort in relation to age was identified with locally estimated scatterplot smoothing (LOESS) (76), and the residuals of the fit were obtained. The width of the moving window relative to the data size, or span, was chosen through iteration by visually examining the dependence of the residuals on age (77). Differences from the same fit were also obtained for the previous and current MdDS cohorts as pseudo-residuals. A between-cohort difference in the distributions of these pseudo-residuals was tested with a two-sample Kolmogorov-Smirnov test. Overall deviations of the pseudo-residuals from zero and between the cohorts were tested with one- and two-sample t-tests, respectively.

We defined a clinically significant improvement, or a success of a treatment in a subject, as the rating on the 0–10 subjective scale of symptom severity being reduced by more than one half of the pre-treatment level (12, 27). Considering the chronicity of MdDS, a symptom score reduction by half or more in an individual was deemed substantial and beyond short-term fluctuations influenced by engaged activities or hormonal changes. Thus, individual success or non-success was defined dichotomously by this criterion. A groupwise success rate was calculated as the ratio of the number of subjects with a significant improvement to the total number of subjects for the immediate post-treatment and follow-up time points. In interpreting a groupwise success rate, we compared it to the outcome of a series of two random draws from the range 0–10. The probability that a second random draw would result in less than one half of the corresponding first draw is smaller than 25%, and therefore, a groupwise probability of success above 25% should represent a strength of a treatment approach. A binomial test was used to determine if this benchmark was statistically achieved.

Interdependence between variables was tested with Spearman's rho. The strength of correlation was interpreted according to a guide suggested for behavioral sciences, such that 0 ≤ |rho| < 0.2 is interpreted as negligible, 0.2 ≤ |rho| < 0.4 as weak, 0.4 ≤ |rho| < 0.6 as moderate, 0.6 ≤ |rho| < 0.8 as strong, and 0.8 ≤ |rho| ≤ 1 as very strong (78).

## Results

### Demographic characteristics

Patients with MdDS were recruited on a rolling basis between April, 2020 through July, 2022. There were 329 applicants, of whom 178 completed all forms with nystagmography reports and met the eligibility criteria on first screening. A total of 45 subjects were enrolled in the study in the order of confirmed eligibility and the condition of being able to be scheduled for the laboratory visits. The remaining candidates were wait-listed for another possible research opportunity if desired. Enrolled subjects were randomly assigned to Group 1 (velocity storage attenuation) or Group 2 (VOR readaptation). Two subjects from Group 2 dropped out of the study by failing to participate in the follow-up—data from the remaining 43 subjects (23 Group 1; 20 Group 2) are reported here. The majority of the subjects were women (83.7%), reflecting the female dominance of the diagnosis (9, 13). Only two subjects from Group 1 and four subjects from Group 2 were locally based, and the majority (86.1%) traveled from outside the New York Metropolitan area to undertake the experimental treatment. The subjects' age ranged from 22 to 78 years old, distributed with characteristics typical of this population (mean, 47.1; SD, 14.0) (9, 13). The durations of the subjects' MdDS episodes ranged from 1 to 90 months and their distribution approximately followed an exponential profile that was positively skewed (mean, 19.9; SD, 22.2). The two groups did not differ in the distributions of sex, age, or MdDS duration (Table 1). All subjects with eye movement recording demonstrated a normal VOR and visual suppression of the VOR.

**Table 1:** Group characteristics.

	Group 1	Group 2	*p*
% women	91.3	75.0	0.22
Age in years, mean (SD)	47.4 (13.9)	46.7 (14.2)	0.87
Duration in months, mean (SD)	19.4 (21.7)	19.8 (24.0)	0.88
g_1_ before, mean (SD)	0.53 (0.13)	0.42 (0.10)	**0**.**005**
g_1_ after, mean (SD)	0.45 (0.14)	0.47 (0.08)	0.74
Tc before, mean (SD)	16.6 (3.9)	15.0 (4.0)	0.22
Tc after, mean (SD)	16.0 (5.4)	15.6 (4.5)	0.79
g_0_ before, mean (SD)	0.102 (0.022)	0.093 (0.030)	0.33
g_0_ after, mean (SD)	0.080 (0.033)	0.099 (0.027)	0.07

Bold typeface indicates *p* < .05.

### Changes in the VOR

Example eye velocity responses to a 60°/s step rotational test, obtained from a single subject from Group 1 on the first and fifth days of the laboratory visits, are illustrated in Figures 2A,B. Estimated contributions of the direct and indirect pathways of the VOR (48) are profiled in the bottom inset of each panel. The pre-treatment characterization of the VOR of all subjects with eye movement recording was such that the mean (SD) g_1_, Tc, and g_0_ were 0.48 (0.13), 15.9 (4.0) s, and 0.098 (0.026). The mean g_1_ was statistically different between the two groups, with that of Group 1 being meaningfully larger [|t(39)| = 2.95, *p* = 0.005, d = 0.93]. As we focused on within-group changes, this unexpected imbalance in g_1_ in the randomly assigned groups presumably did not create an intrinsic bias in the study. The groups did not differ significantly in the pre-treatment Tc or g_0_ (Table 1), i.e., the velocity storage characteristics of the two groups were similar.

**Figure 3:**
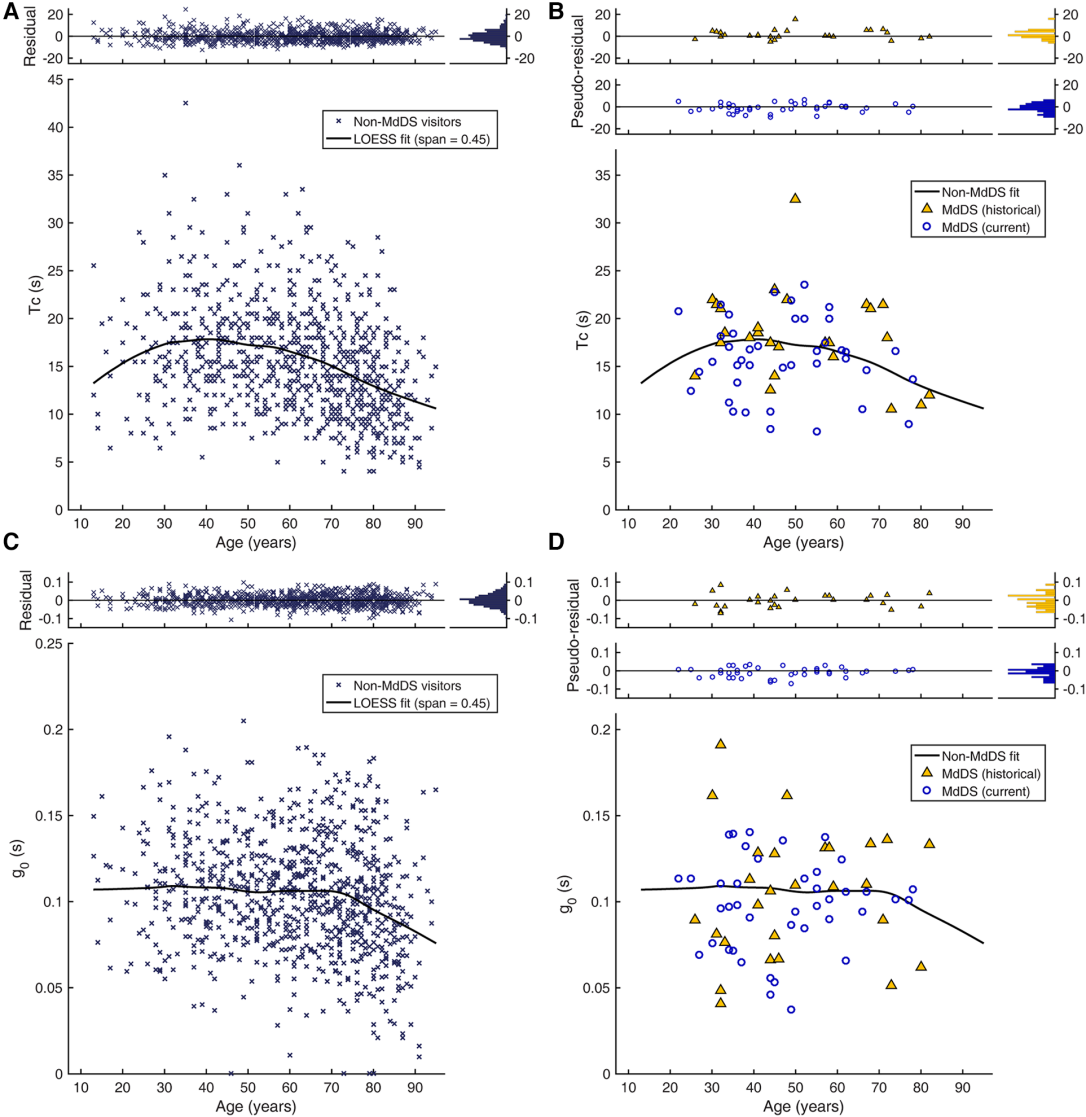
Age dependence of velocity storage parameters. (**A**) Tc of non-MdDS laboratory visitors (*n* = 911). Individual Tc values are plotted against age. The top inset shows the distributions of individual deviations from the trend curve (residuals). (**B**) Tc of two cohorts of patients with MdDS. The top inset shows the distributions of the two cohorts’ individual deviations from the trend curve from (**A**) (pseudo-residuals). (**C**) g_0_ of the non-MdDS laboratory visitors. (**D**) g_0_ of the two patient cohorts.

To the extent that MdDS may be caused by malfunctioning velocity storage, we sought to determine whether the pre-treatment Tc of patients with MdDS was different from those of individuals without MdDS or other vestibular dysfunction known to affect velocity storage. Since age is a known confounding variable (53), the 911 historical data points of non-MdDS laboratory visitors with presumably normal VOR were plotted against age (Figure 3A). The inter-individual variability was large relative to the 4 s time constant fixed for the semicircular canal response. To elucidate the underlying effect of age, the data were fit with LOESS with a span of 0.45. The resulting trend curve was overall convex upward and reached the maximum time constant value of 17.8 s at the age of 41 years. The SD of the residuals was 5.2 s. Given that the residuals did not differ by sex [|t(909)| = 1.11, *p* = 0.27] and that the number of men in the MdDS cohorts was small, comparisons were conducted with both sexes combined. The Tcs of the historical and current cohorts of patients with MdDS obtained before any treatment were then superimposed on the trend curve created for the non-MdDS laboratory visitors (Figure 3B). The inter-individual variability was also large in these cohorts. The two cohorts did differ from each other in the distributions of the pseudo-residuals relative to the trend curve [D(28,41) = 0.334, *p* = 0.038]. The pseudo-residual means (SD) of the historical and current patient cohorts were 1.7 (4.0) s and −1.0 (4.0) s, respectively, and their difference was also statistically significant [|t(67)| = 2.73, *p* = 0.008]. However, only the pseudo-residual mean of the of the historical patient cohort was significantly different from zero [|t(27)| = 2.20, *p* = 0.036], and the effect sizes of the deviations were both small (historical: d = 0.42; current: d = 0.25). Thus, evidence for abnormal Tcs in MdDS was deemed weak.

Similarly, we sought to determine whether the pre-treatment g_0_ was different in patients with MdDS (Figures 3C,D). The LOESS trend curve obtained from the non-MdDS visitors was nearly flat from ages 13 through 70 years old, taking on values of ≈0.107, and thereafter steadily declined with a slope of ≈−0.0013 per year. The residuals statistically significantly differed by sex [|t(909)| = 2.58, *p* = 0.01], with the female mean (SD) 0.002 (0.033) above the trend curve and the male mean −0.004 (0.031), below the curve, but this numerically small difference was deemed not to be practically meaningful (d = 0.18). Therefore, as with Tc, comparisons with the MdDS cohorts were conducted with both sexes combined. The two MdDS cohorts did not differ in the means of the pseudo-residuals [|t(67)| = 0.80, *p* = 0.42] or their distributions [D(28,41) = 0.21, *p* = 0.41]. The combined pseudo-residuals in turn were not statistically different from the residuals of non-MdDS visitors [|t(978)| = 1.48, *p* = 0.14]. Thus, an abnormal g_0_ was also not identified as a characteristic of MdDS.

With the treatment regimen, there was a statistically significant change in the g_0_ of Group 1 [|t(21)| = 3.95, *p* < 0.001], with a mean (SD) reduction by 0.023 (0.027). The effect size of the change was large (d = 0.84). This change is illustrated in Figure 2C (rightmost panel) with the filled circles falling mostly below the identity line drawn diagonally. Although unintended, the change in g_1_ was also statistically significant in Group 1 [|t(21)| = 2.62, *p* = 0.016]. The effect size of this change was medium (d = 0.56). On the other hand, a statistically significant change in Tc was not detected. Thus, the visual-vestibular conflict regimen applied to Group 1 modified velocity storage in patients with MdDS by reducing the coupling gain, but not the rate of charge/discharge, and additionally reduced the gain of the initial fast VOR response. The reductions in g_0_ and g_1_ showed only a weak, statistically non-significant correlation to each other (rho = 0.31, *p* = 0.17) while their correlation to Tc was both negligible.

As expected, no statistically significant change in any of the three VOR parameters was detected for Group 2, which in Figure 2C is illustrated as open triangles falling both above and below the identity line in each panel. Thus, the strength of the velocity storage contribution to the VOR was not systematically affected by the readaptation regimen applied to this group. There was nevertheless some fluidity in the data, and individual changes in Tc measurements showed a moderate but statistically non-significant negative correlation with those in g_0_ (rho = −0.48, *p* = 0.052) and a weak, non-significant positive correlation with those in g_1_ (rh0 = 0.21, *p* = 0.41). The correlation between the changes in g_1_ and g_0_ was negligible.

### Changes in symptoms

**Figure 4:**
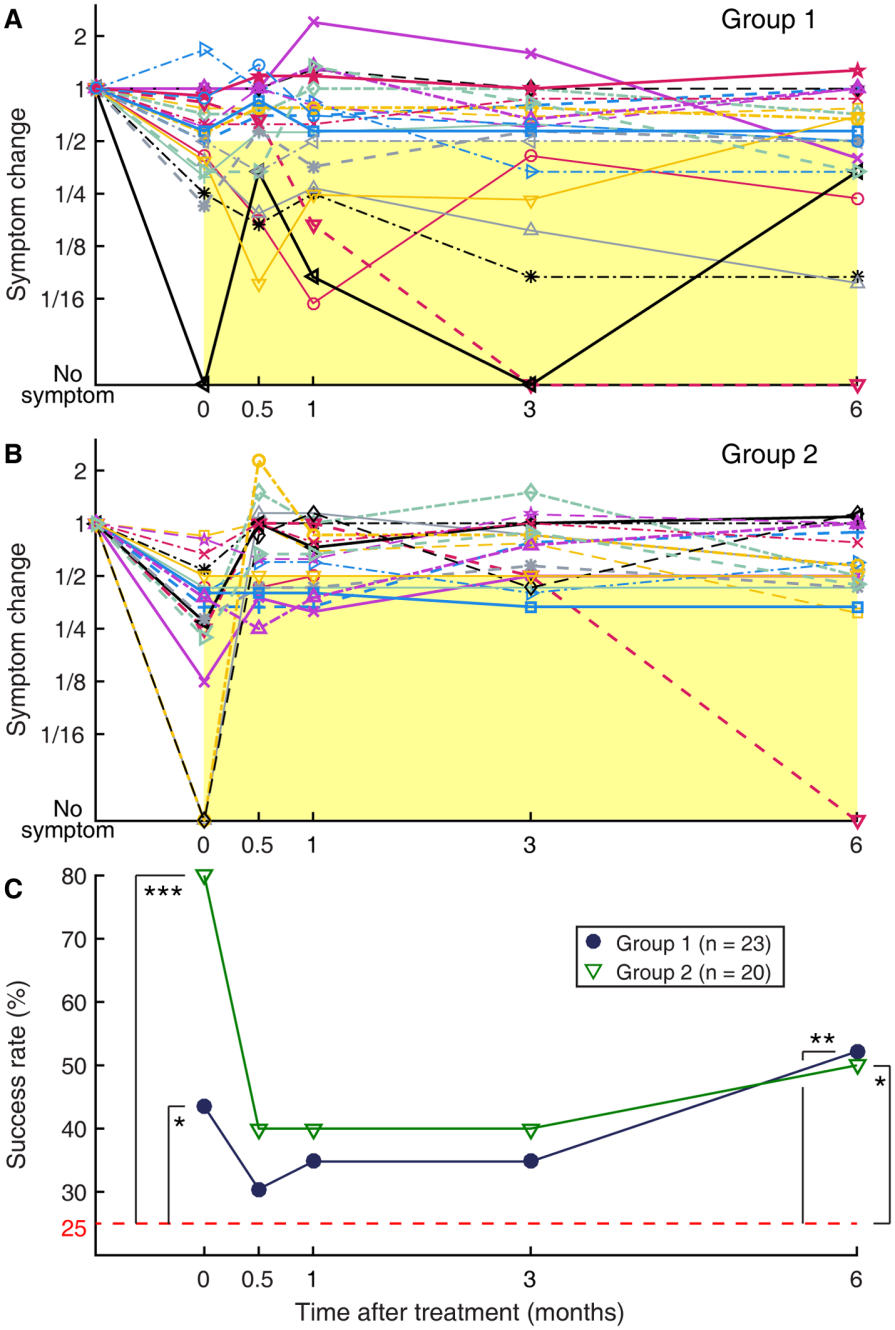
Longitudinal changes in subjective symptom rating, normalized to the pre-treatment level. (**A**) Group 1. (**B**) Group 2. Each set of connected markers indicate an individual subject. Time zero indicates immediately after the treatment. All responses are normalized relative to the pre-treatment symptom level, to which a value of 1 is assigned. Markers falling on the yellow rectangular areas indicate a successful outcome defined as more than a halving of symptom severity relative to the pre-treatment level. (**C**) Summary of (**A**) and (**B**) plotted as groupwise “success” rate over time. Filled circles: Group 1; open triangles: Group 2. The dashed horizontal line indicates the expected rate with random reporting of a halving of symptom severity, i.e., group-wise non-recovery. *p < 0.05. **p < 0.01. ***p < 0.001.

Upon completing the treatment regimen, 19 of the 23 subjects of Group 1 rated their symptoms as having been reduced from the pre-treatment level, of whom 10 reported a reduction by more than half (Figure 4A). Thus, the immediate success rate for Group 1 was 43%, which was above a chance level (*p* = 0.041), indicating a strength of the treatment. This rate is displayed as the left most filled circle marked with an asterisk in the summative figure (Figure 4C). Of the remaining four subjects, three reported no change in their symptoms, and one reported worsening of symptoms. The worsening of symptoms in this subject (Subject 15) was on account of a transient increase in visual sensitivity that occurred on the last two days of the laboratory visits as the visual-vestibular conflict used in the treatment was intensified. For Group 2, all 20 subjects rated their symptoms as having been reduced from the pre-treatment level, of whom 16 reported a reduction by more than half (Figure 4B). Thus, the immediate success rate of the readaptation protocol was 80%, indicating a great strength of this treatment (*p* < 0.001) at a rate similar to those previously reported (12, 45). This rate is displayed as the left most open triangle marked with three asterisks in Figure 4C.

Exposure to passive motion during a long travel to return home after the treatment or during any subsequent occasion was previously noted as a major trigger for symptom recurrence (12, 26). Group 2 was particularly vulnerable to this effect, as evidenced by a trend for symptoms to bounce back in individuals and a corresponding sharp decline in the groupwise success rate at the two-week (0.5 months) post-treatment follow-up assessment (Figures 4B,C). Only five of the 16 subjects with initial success in Group 2 continued to experience more than a halving of symptoms relative to the pre-treatment level throughout the 6-month follow-up period. The other 11 subjects with initial success experienced a symptom rebound at some point during the 6-month follow-up period, including one who reported more than a doubling of symptom rating relative to the pre-treatment level at two weeks post-treatment, although this increase was later partially reversed. In total, there were a total of five subjects with initial success who reported a symptom rebound to the pre-treatment level or worse at two weeks post-treatment, and they were all non-local participants who necessarily were exposed to prolonged passive motion on their way home after the treatment. Three of four local participants in Group 2 were initially successfully treated, and none of the three reported a symptom rebound to the pre-treatment level at two weeks post-treatment. Furthermore, in Group 2, the immediate post-treatment outcome did not predict the long-term outcome; the normalized symptom ratings at two-week through six-month post-treatment were not statistically significantly correlated with the immediate post-treatment symptom rating (|rho| < 0.26, *p* > 0.27) (Table 2).

**Table 2:** Correlation between immediate post-treatment symptom rating and those in longer terms.

		2 weeks	1 month	3 months	6 months
Group 1 (*n* = 22)	rho	**0**.**81**	**0**.**64**	**0**.**58**	**0**.**49**
	*p*	**<0**.**001**	**0**.**001**	**0**.**004**	**0**.**020**
Group 2 (*n* = 20)	rho	−0.20	−0.25	0.06	−0.05
	*p*	0.394	0.278	0.806	0.823

Bold typeface indicates *p* < .05.

Compared to Group 2, and as intended, Group 1 was more resistant to symptom recurrence. Although the overall success rate for this group dropped from 43% to 30% at two weeks post-treatment, none of the 10 subjects with initial success reported a symptom rebound to the pre-treatment level (Figures 4A,C). This result is despite that all these subjects were non-local participants and were exposed to prolonged passive motion on their way home after the treatment. Over the six-month follow-up period, only one of these 10 subjects with initial success reported that the symptoms gradually returned to the pre-treatment level, while 5 subjects reported continuing to experience significantly reduced symptoms throughout the six-month period, and the remaining 4 reported symptom fluctuations but around an overall reduced level. Subject 15, who developed a transient increase in visual sensitivity during the treatment, rated the symptom level at two weeks post-treatment as unchanged from the pre-treatment level. On the other hand, the two local participants in Group 1 turned out not to successfully respond to the treatment, and their symptoms fluctuated during the six-month follow-up period. In general, despite the fluctuations in symptom ratings over time, the immediate post-treatment outcome was predictive of those in longer terms in Group 1. The correlation with the immediate post-treatment symptom rating was very strong at two weeks post-treatment (rho = 0.82, *p* < 0.001), gradually reducing to a weak and statistically non-significant level at six months post-treatment (rho = 0.38, *p* = 0.072). However, when Subject 15 was excluded from the analysis, the correlation remained very strong to moderate and statistically significant throughout the six-month follow-up period (rho > 0.49, *p* < 0.020) (Table 2).

### Relation between VOR characteristics and treatment responsiveness

Finally, we examined whether the responsiveness to the treatment was correlated with the VOR parameters, g_1_, Tc, and g_0_. However, for either group (and for Group 1, with or without Subject 15), the immediate post-treatment change in symptom rating was at best weakly, and statistically non-significantly correlated with either pre- or post-treatment values of g_1_, Tc, or g_0_ (median |rho| = 0.15, *p* > 0.10). Furthermore, despite that Group 1's visual-vestibular conflict regimen, designed to attenuate velocity storage, indeed succeeded in reducing g_0_, there was no clear correlation between this change and the reported symptom change. There was also no clear correlation between changes in g_1_ or Tc and that in symptoms. On the other hand, even though there was no groupwise change in any of the three VOR parameters in Group 2, a moderate correlation was found in Group 2 between reduced g_1_ and reduced symptom rating (rho = 0.57, *p* = 0.016) and between increased g_0_ and reduced symptom rating (rho = −0.51, *p* = 0.036). As changes in g_1_ and g_0_ were not correlated in this group, the implication for these correlations, spurious or not, is not clear.

## Discussion

In this study, we investigated whether symptoms of MdDS could be improved by attenuating the velocity storage contribution in the central vestibular pathways by using a slightly intensified version of a vestibular habituation protocol that was previously developed for motion sickness treatment (58). Because velocity storage is thought to contribute to the perception of spatial orientation and self-motion (28, 30, 31, 39), we reasoned that spatial disorientation and false sensation of self-motion in MdDS might be curbed when the contribution of a presumably malfunctioning velocity storage mechanism was limited with this protocol. A successful outcome defined as a more than halving of the subjective symptom rating from the pre-treatment level was initially achieved in 43% of the 23 subjects who underwent this treatment regimen (Group 1). This rate of success was at an above-chance level and represented a strength of the approach. Given that MdDS was previously considered intractable (3), the treatment regimen, composed of low-frequency oscillation coupled to a conflicting visual stimulus, is a welcome addition to the emerging countermeasures to the illness (12, 22, 26, 38, 79, 80). Remarkably, the initial impact of the treatment was strongly predictive of the long-term outcome, with the majority of positive responders reporting overall reduced symptoms during the 6-month follow-up period. Thus, if initially effective, the treatment also had a long-term prophylactic effect against symptom relapse. This result is consistent with the long-term retention of velocity storage attenuation previously demonstrated in both animals and humans (55, 57, 58, 65).

We found a clear contrast in the long-term outcomes of the two treatment approaches that we delivered, one aimed to attenuate velocity storage (Group 1) and the other to correct the spatial orientation properties of velocity storage (Group 2). The latter, the VOR readaptation regimen, yielded a high initial success rate, presently at 80%, similar to those previously reported (12, 26). However, the initial impact was not predictive of the subsequent symptom reports, supporting that spatial readaptation of the VOR is not prophylactic of symptom relapse, presumably because the treatment regimen does not change the adaptive potential of the velocity storage mechanism.

As expected from the experimental design, systematic changes in the VOR response to a velocity-step rotation were shown only in the group that underwent the modified vestibular habituation protocol (Group 1). The protocol attenuated the contribution of velocity storage by way of a reduction in the coupling gain, g_0_, but not of a reduction in the rate of charge/discharge, Tc. The protocol in addition reduced the gain of the rapid VOR response, g_1_. These outcomes in patients with MdDS are at odds with the original application of the protocol in a motion sickness study involving both healthy normal and motion sickness-susceptible individuals (58). In this study, the velocity storage contribution was also attenuated but by way of a reduction in Tc without a change in the VOR gain. The source of the discrepancy is presently unknown, but the training stimulus was intensified at a faster pace and to a greater degree in the present application of the protocol. In addition, as visual coupling to velocity storage has been reported to be saturated at only ≈20°/s in humans (81), the OKS may have provided incomplete counteraction to the VOR during rotation at the speed used in the present study. Also puzzling is that the induced changes in the VOR parameters, particularly g_0_, did not correlate with those in symptom rating even though attenuation of velocity storage was hypothesized to cause symptom improvement. This disconnection may be because of the multifacetedness of MdDS symptomatology and individual differences in the emphasis of various symptoms when reporting the overall symptom severity. Furthermore, since the VOR was tested only during the laboratory visits, how long the changes in the VOR parameters were retained is unknown. However, g_1_ presumably would have been recalibrated quickly in a natural environment independently of the velocity storage parameters (57).

What determines the natural strength of velocity storage contribution to the VOR is not well understood (59–62, 64, 82), but age is a known mediating factor such that Tc increases through early adulthood and transitions in middle adulthood toward a decrease (52, 53). We confirmed this general trend in a large data set from a historical cohort consisting of individuals without MdDS or other vestibular dysfunction known to affect velocity storage. An earlier study provided a slightly longer estimate of vestibular time constant peaking at a slightly younger age (53). However, these variations may be explained by the differences in the test paradigms, assumptions regarding the underlying structure of the response, or age distributions of the samples, whether the age-based fit had an assumed shape or was data-driven, or any combination of these or other factors. The data from the historical cohort further indicated that age might also mediate g_0_, but unlike for Tc, the data-driven fit indicated stability of g_0_ over much of the age span followed by a decline in senescence. The implications of these findings, in terms of functional consequences or mechanistic bases, are presently unclear.

Against this backdrop, we found no evidence to associate MdDS with abnormal Tc or g_0_. That is, a naturally long Tc or high g_0_ does not appear to be a risk factor for MdDS, nor does a naturally short Tc or low g_0_ appear to have a prophylactic effect. There is a strange juxtaposition between this conclusion and the results that, while training with the velocity storage attenuation regimen was associated with symptom improvement and a possible prophylactic effect, a direct association between the observed training-induced reduction of g_0_ and symptom improvement was not evident. Further, the VOR parameters we studied were not predictive of the treatment responsiveness in either group. Lastly, even though we expected the VOR readaptation regimen to change the orientation properties of velocity storage without changing Tc or g_0_, and we indeed found no group-wise systematic change in these parameters, the interindividual variations in the Tc and g_0_ changes were moderately anticorrelated. This unexpected relation may be a reflection of a complexity arising from reshaping the three-dimensional structure of velocity storage. These unsolved problems highlight that understanding malleability of velocity storage and its consequences is an important research direction.

A practical clinical implication of this study is that a therapy technique aimed at attenuating velocity storage shows promise as a lasting remedy for MdDS that can complement the VOR readaptation approach (12, 26). We cannot completely rule out the possibility of a placebo effect because treatments in our laboratory are now highly sought after, and patients may have arrived with higher expectations than other treatments that they had tried previously. Nevertheless, the contrast between the outcomes of the two approaches in both the immediate and long terms is in support of a true clinical effect. Although the VOR readaptation approach is gaining recognition as being effective, the risk of relapse may make the treatment most useful when conducted at clinics local to patients (41–44) or through telemedicine using a portable device (27). However, a significant roadblock associated with the VOR readaptation approach currently is the availability of resources and clinical expertise required for determining the stimulus parameters. On the other hand, the regimen we used in this study to attenuate velocity storage followed a rigid protocol with little interpersonal variation. Velocity storage can also be attenuated with a simple protocol that uses a large repetition of rotations in darkness or with the eyes covered, albeit perhaps with a different efficiency (55, 65). Therefore, attenuation of velocity storage is a pragmatic clinical option in the treatment of MdDS. It remains to be tested whether combining this approach with VOR readaptation, when achievable, can yield a high probability of success with robust long-term benefits.

## Data Availability

The datasets presented in this article are not readily available because the raw data were collected using custom-made software. However, the datasets will be made available by the author upon reasonable request. Requests to access the datasets should be directed to SY, sergei.yakushin@mssm.edu.
